# The Role of Vaccine Coverage within Social Networks in Cholera Vaccine Efficacy

**DOI:** 10.1371/journal.pone.0022971

**Published:** 2011-07-29

**Authors:** Elisabeth D. Root, Sophia Giebultowicz, Mohammad Ali, Mohammad Yunus, Michael Emch

**Affiliations:** 1 Department of Geography and Institute of Behavioral Sciences, University of Colorado at Boulder, Boulder, Colorado, United States of America; 2 Department of Geography and Carolina Population Center, University of North Carolina at Chapel Hill, Chapel Hill, North Carolina, United States of America; 3 Data Management, GIS, and Statistics Unit, International Vaccine Institute, Seoul, Korea; 4 ICDDR,B, Centre for Health and Population Research, Dhaka, Bangladesh; Umeå University, Sweden

## Abstract

**Background:**

Traditional vaccine trial methods have an underlying assumption that the effect of a vaccine is the same throughout the trial area. There are, however, many spatial and behavioral factors that alter the rates of contact among infectious and susceptible individuals and result in different efficacies across a population. We reanalyzed data from a field trial in Bangladesh to ascertain whether there is evidence of indirect protection from cholera vaccines when vaccination rates are high in an individual's social network.

**Methods:**

We analyzed the first year of surveillance data from a placebo-controlled trial of B subunit-killed whole-cell and killed whole-cell-only oral cholera vaccines in children and adult women in Bangladesh. We calculated whether there was an inverse trend for the relation between the level of vaccine coverage in an individual's social network and the incidence of cholera in individual vaccine recipients or placebo recipients after controlling for potential confounding variables.

**Results:**

Using *bari*-level social network ties, we found incidence rates of cholera among placebo recipients were inversely related to levels of vaccine coverage (5.28 cases per 1000 in the lowest quintile vs 3.27 cases per 1000 in the highest quintile; p = 0.037 for trend). Receipt of vaccine by an individual and the level of vaccine coverage of the individual's social network were independently related to a reduced risk of cholera.

**Conclusions:**

Findings indicate that progressively higher levels of vaccine coverage in *bari*-level social networks can lead to increasing levels of indirect protection of non-vaccinated individuals and could also lead to progressively higher levels of total protection of vaccine recipients.

## Introduction

Epidemiological theory is founded on the assumption of “homogeneous mixing,” with susceptible and infectious individuals mixing uniformly, without regard to age, location or other factors [1], [2]. However, a number of spatial and behavioral factors alter the rates of contact among infectious and susceptible individuals, which has implications for vaccination strategies and the evaluation of vaccination campaigns [2]. If susceptible individuals come in contact more often with immunized individuals than with non-immunized (possibly infected) individuals, they are less likely to contract the disease. Thus, a person's contact network is an important determinant of disease transmission as is the level of vaccination within that contact network [3]–[5]. Traditional vaccine trial methods typically evaluate the effectiveness of a vaccine by examining population-level morbidity and mortality, essentially ignoring possible heterogeneity due to differences in exposure among individual contact networks. To address this limitation, several new methods for evaluating vaccine efficacy have been developed. *Ecological vaccine trials* incorporate geographic information so that analysis of vaccine effectiveness can be conducted on geographic subpopulations [6], [7]. The premise behind a geographic analysis is that individuals are more likely to interact with others who are closer to them in space than those located further away [8]. Thus, disease and vaccination rates within a specific geographic area (often referred to as a “neighborhood”) represent exposure from an individual's contact network. *Contact network epidemiology* and *social network analysis* provide new methods for modeling the patterns of interactions among individuals that can lead to the transmission of an infectious disease [9]–[11]. These methods assume that individuals who are socially connected through kinship, friendship or work interact more often than those who are not [9]. Social network analysis is used to measure relationships between social entities [12], [13] and is particularly useful for measuring social relationships that influence disease outcomes or health interventions [9]. The probability of becoming infected is therefore conditioned by the number of infected and vaccinated individuals within a social (or contact) network.

In previous research, we used ecological vaccine trial methods to examine the geographic variation in the efficacy of a cholera vaccine. Analyses were conducted using results from a community-based individually randomized oral cholera vaccine trial conducted in Matlab, Bangladesh in 1985. Initial results suggested a protective efficacy (PE) of only ∼50% [14]. Our reanalysis of trial data found significant spatial heterogeneity in PE and suggested this spatial variation was due to ecological differences and/or spatial variation of vaccinated individuals in the study area [15]. Results also illustrated that variation is inversely related to vaccine coverage (i.e.,% of people vaccinated in an area) after adjusting for several ecological factors [7]. Higher levels of neighborhood vaccine coverage were linked to lower risk of cholera among residents, both in placebo recipients, for whom a strong inverse relationship was observed, and in vaccinees, for whom a suggestive relationship is evident [16]. These findings are consistent with the concept of herd immunity, which occurs when vaccination of a group of individuals in proximity to one another reduces the intensity of transmission of the infection among all members of the group regardless of immunization status [6]. The cholera vaccine conveyed a certain amount of “direct” protection, which is the protection conferred to a vaccinated individual because of biological immunity. But, progressively higher levels of vaccine coverage also appeared to convey higher levels of indirect protection of non-vaccinees in addition to the direct protection of vaccines [7], [16].

While our prior research used spatially-defined neighborhoods to model potential fecal-oral contact and the effect of the cholera vaccine, we hypothesize that social connectedness is also likely to influence disease transmission because contact networks are often determined by social interactions. This study adapts techniques from the social network and contact network epidemiology literature; we use kinship-based social networks to better model individual exposure to vaccinated individuals and potentially contaminated food and water. The *V. cholerae* pathogen is spread through the fecal-oral route but can also survive naturally in seas, ponds, and other aquatic environments [17], [18]. Thus, two modes of transmission based on these reservoirs have been identified. *Primary transmission* occurs via the local estuarine environments where *V. cholerae* is able to survive, spreading to the individual through some form of contact with water or, alternatively, consumption of shellfish or aquatic plants contaminated in their local habitat. *Secondary transmission*, in turn, refers to the diffusion of cholera from an infected individual to susceptibles in the population through fecal contamination. Secondary transmission occurs through person-to-person contact that is driven by human interaction and social contact, which leads to contamination of shared water sources [19]–[22]. The occurrence of direct person-to-person transmission is considered rare by some and supported by others [23]–[25]. In this study, we focus on the role of secondary transmission. If social contact is an important factor in the transmission of cholera and the efficacy of the vaccine, we would expect to see lower overall cholera rates and lower placebo group incidence among individuals with a high level of vaccination in their social network.

Past vaccine trials have not stratified placebo incidence or efficacy by social network connectivity because the studies did not collect information on social connections. This paper further analyzes this vaccine trial by exploring the effect of kinship-based social networks on protective efficacy and cholera risk. We hypothesize that protective efficacy and placebo group incidence are influenced by social networks because herd immunity is affected by the level of vaccination within an individual's social (or contact) network. Thus, unvaccinated people who are socially connected to people who have been vaccinated will be at lower risk for cholera due to social interactions that lead to less contaminated food and shared water environments (e.g., ponds, latrines) and because they are less likely to be exposed to the disease. By taking a social network approach, this research contributes to the discussion of how to plan, conduct and evaluate vaccine trials and will also provide insight into cholera transmission dynamics.

## Methods

### Study Area

The cholera vaccine trial was conducted in Matlab, Bangladesh, the research site of ICDDR,B. Matlab is located approximately 50 km south-east of Dhaka at the confluence of the Meghna and Ganges Rivers. Cholera is endemic in this region of Bangladesh. Rural Bangladeshis live in *baris*, which are groups of patrilineally-related households. *Baris* are located on raised plots of land, which typically resist flooding during monsoon season, and are surrounded by agricultural fields. An average of six distinct households constitute a *bari* and the different households in a *bari* are typically comprised of related individuals.

### Data

This study uses two datasets including the original cholera vaccine trial database and a longitudinal demographic database from which the vaccine trial participants were selected.

#### Cholera Vaccine Trial Data

Details of the vaccine trial and database are documented comprehensively elsewhere [14], [16]. Briefly, a community based individually randomized oral cholera vaccine trial was conducted in Matlab in 1985. This double-blind trial measured the efficacy of two vaccines, the B subunit-killed whole cell (BS-WC) and the killed whole cell (WC) only vaccines. The control agent was *E. coli* K12 strain. Females aged 15 years and older and children aged 2–14 were the target groups of the trial. Three vaccine doses were given at 6 week intervals to 62 285 people in the target group. Identification of cholera cases took place at one hospital in Matlab and two community-based treatment centers in the study area. During 5 years of follow up, the cumulative protective efficacy was 49% in the BS-WC group (p<0.001) and 47% in the WC group (p<0.001) [26]. Protection by each vaccine was evident only during the first three years of follow-up and was lower in children who were vaccinated at 2–5 years then in older persons [26]. Because the two types of the cholera vaccines were identical in composition, apart from the inclusion of the B subunit, and because they conferred similar levels of protective efficacy [16], we grouped recipients of these vaccines together for analysis. The efficacy calculations are based on cholera cases that occurred between 14 and 365 days after a second dose of the placebo or vaccine. Because population migration progressively changed the composition of households and *baris* after dosing and because our aim was to investigate cholera incidence within stable social networks, which were developed using migration data, we chose to restrict our analyses to the first year of follow up after dosing. Such a short interval allowed us to assume the household populations and social networks were stable.

#### Longitudinal Demographic Data

Vaccine trial data were linked to the Matlab Health and Demographic Surveillance Systems (MHDSS) using a person-specific unique ID. The MHDSS is the most comprehensive longitudinal demographic database of a large population in the developing world and has recorded all vital demographic events and internal migrations of the study area population since 1966. In addition, people are visited monthly by community health workers and if they have severe diarrhea are treated at a hospital run by ICDDR,B. The MHDSS was used to associate known risk factors, such as age, sex, and religion, with individuals in the vaccine trial database and to construct the kinship-based social networks.

Kinship networks were created two different ways: using household-level and *bari*-level kinship connections. Household-level connections offer more specificity about kinship social ties while *bari*-level connections may capture a larger social network that includes non-related individuals that live nearby. The HDSS maintains all kinship ties among the Matlab residents and the exact dates each person resided in a household over time. Therefore, an individual can be traced from household to household over the course of his or her life (as long as he or she resides in the Matlab study area). Each individual in the HDSS has three identification (ID) numbers: a registration ID (RID), their current ID (CID) and a *bari* ID. When an individual is born or moves into the study area they are assigned a RID, which does not change during their lifetime. If a person moves into a new household, almost exclusively due to marriage, they are assigned a new CID and, if that household is located in a different *bari*, a new *bari* ID. This combination of ID numbers can be used to create networks of related individuals.

The kinship-based social networks we use in this study are based on individual-level migrations linking households or *baris*. We assume that when an individual moves to a new household, s/he maintains contact with the previous household or *bari* of residence. The mutual interaction between the old and new households forms a non-directional social connection. Each individual-level migration from household x to household y creates a social linkage between those two households and the *baris* in which they are located. Each linkage of this type is called a dyad. A complete list of all dyads, or an edgelist, can be represented as an *n* x *n* matrix, where *n* equals the number of households.

**Figure 1:**
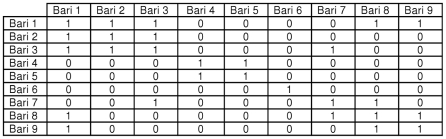
Matrix network example of *bari*-level kinship social connections. GREY circles indicate *baris* while RED lines indicate a kinship-based social connection between the two linked *baris*.

A network matrix is a rectangular arrangement of a set of elements represented as cells that are organized within rows and columns. These matrices allow mathematical and computational tools to summarize and find patterns [12], [13]. Figure 1 shows a hypothetical matrix of kinship relationships between *baris* 1 through 9. In a social adjacency matrix, 1 represents the presence of a single, non-directional social connection between two *baris* and 0 represents no social connection. In Figure 1, a value of 1 is given if there is a kinship relationship, while a value of 0 denotes that there is no relationship. Note that individuals in a *bari* can have kinship ties with other individuals in the same *bari,* which is shown in the table by a value of 1 given to the relationship between a *bari* and itself.

**Figure 2:**
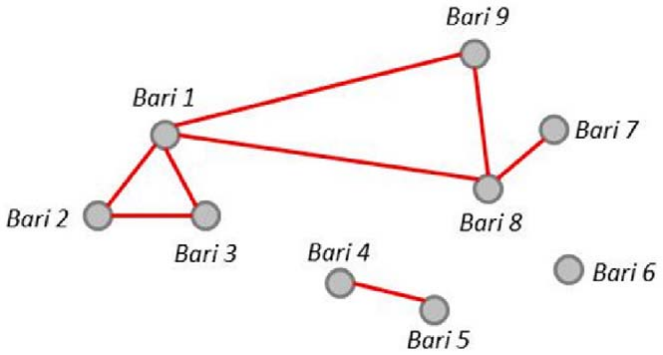
Graph network example of *bari*-level kinship social connections. A “0” indicates no social connection between two baris while a “1” indicates a kinship-based social connection exists.

We can also represent kinship relationships as graphs, which is another form of visualizing networks. Figure 2 is the graph representation of the matrix shown in Figure 1. The nine *baris* included in Figure 2 show the kinship ties between all *baris*. Individuals in b*ari* 6 have no external kinship ties (though individuals within the *bari* are tied to each other). Individuals in *baris* 1, 2, and 3 are related, and individuals in *bari* 1 have kinship ties to two additional *baris* (9 and 8). Individuals in *bari* 8 are related to *bari* 9 and 7, as are individuals in *baris* 4 and 5. Both the network matrix and graph can be built for kinship ties based on *baris* (as shown here) and households.

### Statistical Methods

PE and vaccination coverage levels were measured by social network. We calculated where there was in inverse, monotonic trend for the relation between the level of vaccine coverage in a social network and the incidence of disease in individuals (vaccinees or placebo recipients) by calculating Spearman's correlation coefficients for quintiles of baris and households ordered according to the level of vaccine coverage. Only baris and households with at least one vaccinee or placebo recipient were ordered into quintiles (some inequalities in the number of individuals in the different quintiles occurred because baris or households, rather than individuals, were grouped). Separate analyses were conducted for household-based and bari-based kinship networks in order to compare cholera risk and protective efficacy calculated using the two different definitions of social network. Thus, the actual level of vaccine coverage differs for the quintiles based on household-level vaccine coverage vs. bari-level coverage. We assessed vaccine protective efficacy as [(1-relative risk of cholera in vaccinees vs. placebo recipients) x 100%] and calculated p values with the *Χ* ^2^ test and 95% CIs with test-based methods.

To estimate the variation in risk of a cholera event associated with the level of vaccine coverage in an individual's social network, odds ratios (ORs) and 95% confidence intervals (CIs) were calculated using generalized estimating equations (GEE) with a logit link function. These models are built using independent and exchangeable within-network correlation matrices to control for the correlation that may exist between individuals in the same social network. Several additional variables known to be associated with the risk of cholera were included to control for potential confounding effects: age, sex, religion, distance from the *bari* of residence to the nearest river and treatment center, occurrence of dysentery during follow-up and receipt of vaccine vs. placebo. We assessed effect modification between receipt of vaccine versus placebo and level of cholera vaccine coverage in an individual's social network as predictors of the risk of cholera as a two-way interaction between these variables in models that contained these variables as main effect terms. Two sets of models were estimated; one which included the level of vaccine coverage calculated using *bari*-level social networks and one which included vaccine coverage calculated using household-level social networks.

## Results

49,336 vaccine recipients and 24,667 placebo recipients were included in our analysis. Within social networks developed using *bari* links, vaccine coverage ranged from 0 to 100% with a mean of 39% and a standard deviation of 15%. Vaccine coverage within social networks developed using household links was similar, ranging from 0 to 100% with a mean of 40% and a standard deviation of 28%. Within a year of vaccination, 204 cholera cases were detected, 96 (47%) of whom had been vaccinated.

**Figure 3:**
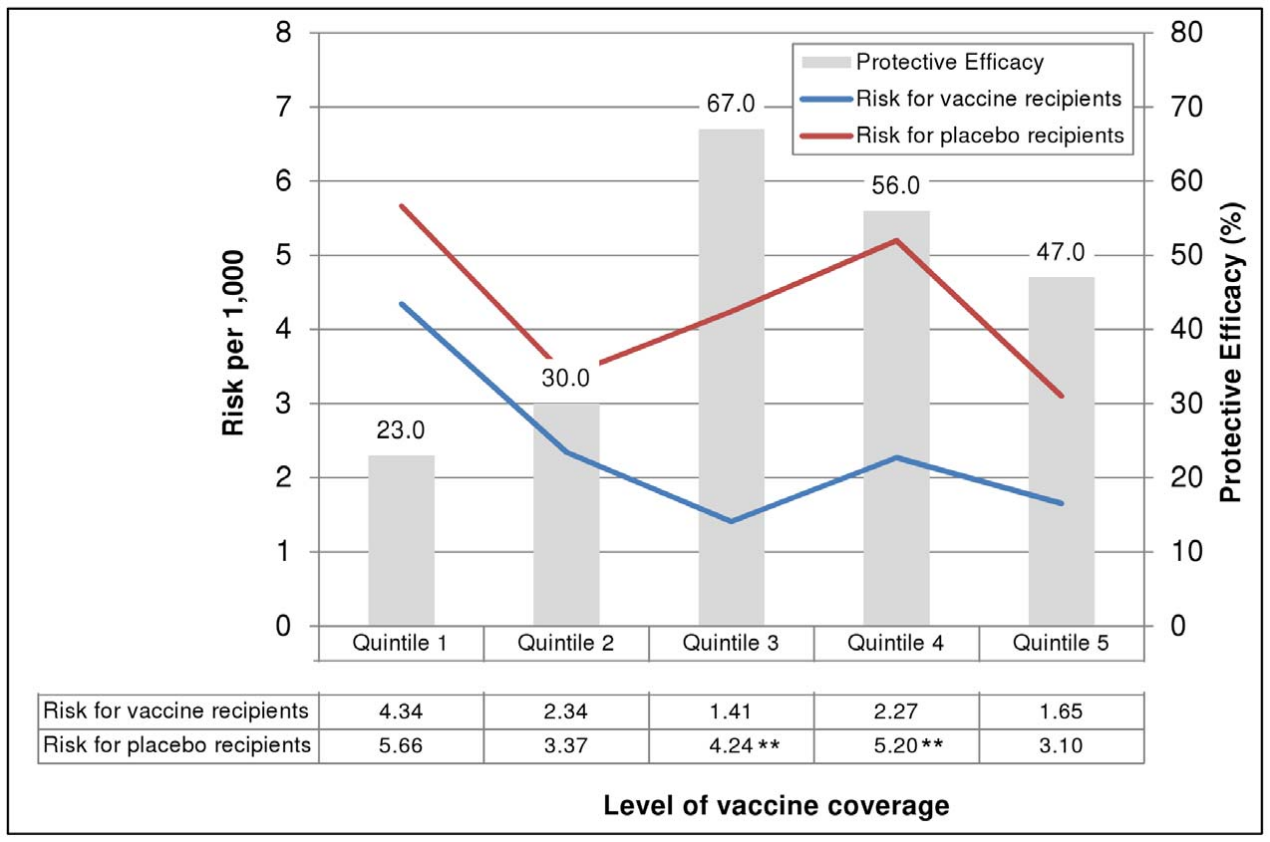
Risk of cholera and protective efficacy of killed oral cholera vaccines, by level of vaccine coverage in household-level social networks. ** p<0.01 for the difference in risk between vaccinees and placebo recipients. Note: Quintile values are as follows: <27.2%, 27.2-40.0%, 40.1-50.0%, 50.1-62.5%, >62.5%. GREY bars show vaccine protective efficacy by quintile of vaccine coverage within social networks developed using household-level kinship connections. The number shown above each bar is the calculated protective efficacy. The RED line indicates the risk of cholera for placebo recipients while the BLUE line indicates the risk of cholera for vaccine recipients by quintile of vaccine coverage within social networks. The numbers contained in the table below the graph indicate the calculated cholera risk for each group. An asterisk (**) indicate that the cholera risk per 1,000 was significantly different between the placebo and vaccine groups (e.g., the confidence intervals for the two calculated rates did not overlap). Quintiles show the proportion of a person's social network that was vaccinated (e.g., for quintile 1, <27.2% of people in an individual's social network were administered the cholera vaccine).

**Figure 4:**
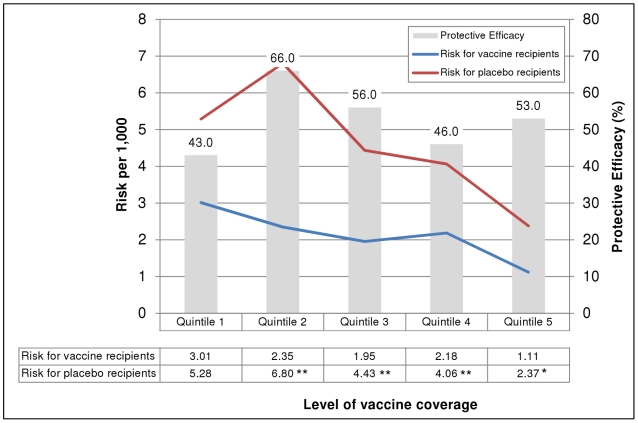
Risk of cholera and protective efficacy of killed oral cholera vaccines, by level of vaccine coverage in *bari*-level social networks. **p<0.01 for the difference in risk between vaccinees and placebo recipients; * p<0.05 for the difference in risk between vaccinees and placebo recipients. Note: Quintile values are as follows: <28.7, 28.7-37.9%, 38.0-44.9%, 45.0-51.8%, >51.8%. GREY bars show vaccine protective efficacy by quintile of vaccine coverage within social networks developed using bari-level kinship connections. The number shown above each bar is the calculated protective efficacy. The RED line indicates the risk of cholera for placebo recipients while the BLUE line indicates the risk of cholera for vaccine recipients by quintile of vaccine coverage within social networks. The numbers contained in the table below the graph indicate the calculated cholera risk for each group. An asterisk (**) indicate that the cholera risk per 1,000 was significantly different between the placebo and vaccine groups (e.g., the confidence intervals for the two calculated rates did not overlap). Quintiles show the proportion of a person's social network that was vaccinated (e.g., for quintile 1, <28.7% of people in an individual's social network were administered the cholera vaccine).

The risk of cholera in recipients of two or more doses of either vaccine or placebo was inversely related to the level of vaccine coverage of the household-level social network (figure 3), though this trend was not significant in placebo recipients (Spearman's correlation coefficient −0.6, p = 0.285) or vaccine recipients (Spearman's correlation coefficient −0.7, p = 0.188). The risk of cholera was also inversely related to the level of vaccine coverage of the *bari*-level social networks (figure 4). This trend was significant for both the vaccine and placebo recipients (Spearman's correlation coefficient −0.9, p = 0.037 for both). In addition, the risk of cholera was significantly lower among vaccinees in the second through fifth quintile of vaccine coverage but this difference was only observed with social networks developed using the *bari*-level connections.

Tables 1 and 2 show the relationship between vaccine coverage of the household-level and bari-level social networks in models that used GEEs with the logit link function that controlled for potential confounding demographic variables known to be associated with risk for cholera in Matlab. Table 1 presents the models that used level of vaccine coverage developed using the *household-level* social networks. In the model examining both vaccine and placebo recipients (model 1), vaccination of the individual and the level of vaccine coverage of the individual's social network were shown to have independent protective effects on an individual's risk of cholera. The inverse relationship between the level of vaccine coverage of the social network and an individual's risk of cholera remained significant in the model that examined only vaccine recipients (model 2: p = 0.006) but not in the model that assessed only placebo recipients (model 3: p = 0.151).

**Table 1:** Predictors of cholera risk in recipients of vaccine or placebo, household-level social networks.

	Model 1: All recipients of > = 2 doses (n = 74,003)			Model 2: Recipients of > = 2 doses of vaccine (n = 49,336)			Model 3: Recipients of > = 2 doses of placebo (n = 24,667)		
	OR*	95% CI	*p*	OR*	95% CI	*p*	OR*	95% CI	*p*
Age	0.98	0.96-0.99	0.0007	0.95	0.92-0.98	0.003	0.99	0.98-1.01	0.181
Sex	1.14	0.84-1.55	0.390	1.18	0.78-1.79	0.421	1.05	0.69-1.61	0.817
Religion	1.15	0.72-1.81	0.563	1.19	0.62-2.29	0.602	1.10	0.60-1.99	0.766
Distance to nearest river	0.86	0.76-0.98	0.025	0.83	0.69-1.00	0.051	0.89	0.75-1.06	0.198
Distance to nearest treatment center	1.11	1.03-1.20	0.005	1.13	1.03-1.25	0.014	1.09	0.99-1.21	0.089
Experienced dysentery	4.30	1.19-15.5	0.026	5.97	1.40-25.44	0.016	3.15	0.47-21.07	0.236
Received > = 2 doses (vaccine vs. placebo)†	0.52	0.39-0.70	<0.0001						
Level of vaccine coverage in network (%)	0.99	0.98-0.99	**0.003**	0.99	0.97-0.99	**0.006**	0.99	0.98-1.00	0.151

*Multivariate odds ratio for the cited variable, adjusted for all other variables in the table.

†Variable was not considered in models 2 and 3 since all individuals were either vaccinated or not in these models.

**Table 2:** Predictors of cholera risk in recipients of vaccine or placebo, *bari*-level social networks.

	Model 1: All recipients of > = 2 doses (n = 74,003)			Model 2: Recipients of > = 2 doses of vaccine (n = 49,336)			Model 3: Recipients of > = 2 doses of placebo (n = 24,667)		
	OR*	95% CI	*p*	OR*	95% CI	*p*	OR*	95% CI	*p*
Age	0.98	0.97-0.99	0.001	0.95	0.92-0.98	0.003	0.99	0.98-1.00	0.202
Sex	1.14	0.83-1.56	0.426	1.18	0.77-1.80	0.443	1.05	0.68-1.62	0.813
Religion	1.11	0.70-1.74	0.664	1.19	0.62-2.29	0.597	1.06	0.58-1.93	0.858
Distance to nearest river	0.88	0.76-1.01	0.004	0.86	0.71-1.03	0.104	0.91	0.75-1.10	0.324
Distance to nearest treatment center	1.12	1.04-1.21	0.065	1.14	1.03-1.26	0.009	1.11	0.99-1.24	0.068
Experienced dysentery	4.63	1.41-15.14	0.011	6.12	1.51-24.85	0.011	3.17	0.46-21.87	0.242
Received > = 2 doses (vaccine vs. placebo)†	0.46	0.35-0.60	<0.0001						
Level of vaccine coverage in network (%)	0.98	0.97-0.99	**0.0002**	0.97	0.96-0.99	**0.003**	0.98	0.97-0.99	**0.008**

*Multivariate odds ratio for the cited variable, adjusted for all other variables in the table.

†Variable was not considered in models 2 and 3 since all individuals were either vaccinated or not in these models.

Table 2 presents the models that used level of vaccine coverage developed using the *bari-level* social networks. In the model examining both vaccine and placebo recipients (model 1), vaccination of the individual showed a significant protective effect on cholera risk (p<0.0001) as did level of vaccine coverage in the social network (p = 0.0002). The inverse relationship between the level of vaccine coverage of the bari-level social network and an individual's risk of cholera remained significant in the model that examined only vaccine recipients (model 2: p = 0.003) and in the model that examined placebo recipients (model 3: p = 0.008).

We did not find evidence of effect modification between receipt of vaccine versus placebo and level of cholera vaccine coverage in an individual's social network. Interaction terms between these two variables were not found to be significant in multivariate models (p = 0.33 for household-level network models and p = 0.65 for *bari*-level network models).

## Discussion

When social networks were built using household-level kinship ties, high levels of cholera vaccine coverage in the social network were linked with a reduced risk of cholera in individuals who received the vaccine, but not for individuals who received a placebo. This shows a direct effect of the vaccine in preventing cholera. However, when social networks were built using *bari*-level kinship ties, high levels of vaccine coverage were linked with reduced cholera risk in *both* vaccinees and placebo recipients. Why the difference between the two social networks? The kinship networks developed using household-level connections represent interactions between households with one or more related individuals. There are, however, households in a *bari* that have no kinship ties. In addition, household with a kinship tie to a household in a different *bari* are only tied to that one household, not to any of the other households in that different *bari*. Thus, household-level social networks do not capture the additional social interactions that occur among individuals from unrelated households located in the same *bari* or unrelated households located in a different *bari* that may be visited while visiting relatives. The *bari*-level kinship networks represent a much larger social network. As long as some kinship tie between two households exists (in the same or a different *bari*) all individuals in the *bari*, regardless or household, are included in the social network. This represents a larger body of social interactions that may include neighbors and friends of related household members. Our findings suggest that including neighbors as well as kinship ties more accurately models cholera transmission dynamics. This is more than likely due to the integral role the environment plays in fecal-oral disease transmission and indicates that shared water and sanitation environments are more important for transmission dynamics than social contacts.

Our results may also be biased due to the fact that social networks were defined only through kinship linkages, which is a limitation of the study. Due to limitations with the dataset, additional network linkages based on non-kinship relationships were not explored. Friends and neighbors were only included in an individual's social network in as a result of being in a *bari* identified by a kinship linkage. Social interaction in Matlab occurs within or outside the household and with acquaintances and neighbors. However, contact with family members and kin is more common, many of which reside within the same household or in a location nearby. The basic social structure of rural Bangladeshi society is anchored in a system of kinship relations [27] and family remains an important institution despite increasing modernization. Activities in daily life, such as labor and meals, often take place in the presence of related individuals [28], [29]. Rural areas also more likely adhere to traditions such as *purdah*, the confinement of women to the home, limiting female social contact to family members [30]. Most secondary transmission of cholera in Matlab likely occurs with food and water acting as vehicles of transmission [31], [32]. If cholera is spread via consumption of water or food contaminated by others, there is a significant chance that the transmission is within the family. Recent research in Matlab suggests that siblings and parents of cholera patients have a higher risk of cholera and that household specific factors (socioeconomic status and hygiene practices) are important determinants of cholera risk [33]. Due to these important social and cultural factors, we hypothesized that kinship-based networks would accurately capture a majority of social interactions that might lead to secondary transmission and contamination of shared water and food. Our findings, however, do not support this hypothesis. Instead we found evidence that cholera transmission was lower, and herd immunity greater, for networks that also included neighboring *baris*.

Further evidence to support our findings regarding transmission dynamics emerged from our previous analyses of the Bangladesh trial that showed that higher levels of vaccine coverage within a 2km spatial neighborhood reduced cholera incidence within the placebo group as well as the vaccine group [7]. This paper employed ecological vaccine trial methods and used a 2 km spatial neighborhood to capture interactions within kinship-based social networks as well as neighbors that may share same water resources. The current analysis adds to previous findings by separating the effects of the social network (included in the 2 km spatial neighborhood and the household-level social network) from the added effects of the local environment (only included in the 2 km spatial neighborhood). The *bari*-level kinship network represents the “in between” case as it captures kinship social networks and some (though not all) neighbors who may share same water resources.

Given this, when the *bari*-level social network is used, findings indicate that progressively higher levels of vaccine coverage in a social network can lead to increasing levels of indirect protection of non-vaccinated individuals and could also lead to progressively higher levels of total (indirect plus direct) protection of vaccine recipients. This finding is similar to previous analyses that used the 2 km spatial neighborhood. When household-level social networks were used, higher levels of vaccine coverage did not lead to high levels of indirect protection of non-vaccinated individuals. This suggests that the local environment plays a *more important role* in the herd immunity effect observed in prior studies and the network based on purely social ties does not capture the role of the environment. The population-based social network does not perform as well as the distance-based social network for the transmission of cholera; therefore models indicate no reduction of the cholera risk with the increase of the coverage among social contacts. Both social ties and the local environment are important for understanding vaccine efficacy and the risk for contracting cholera.

While there is a small but well-regarded body of prior literature examining the influence of social networks on diseases that have a behavioral component such as STIs and obesity [34]–[37], few studies have examined the effect of social ties on infectious disease transmission. Giebultowicz, et al [38] found that the rate of cholera in an individual's social network was an important factor in determining cholera risk, but that the neighborhood environment was of greater importance in predicting higher rates of disease. Their paper provided important information on cholera transmission dynamics but *did not investigate the impact of vaccination within a social network on disease rates*. In Ecuador, Bates et al. [39] found that high levels of social connectedness and centrality were risk factors for diarrheal disease, though disease incidence among those connections was not considered in the analysis. We are not aware of any studies that examine the role of social networks in vaccine efficacy. In contrast to sexually transmitted infections, the study of enteric disease transmission requires an understanding of all social connections (not just sexual contacts) and accurate characterization of the shared water environment. The protective efficacy of a vaccine may, therefore, be dependent on the level of vaccine coverage within an individual's social network *as well as among individuals in the surrounding neighborhood.* Our data suggests that the environment and social interactions within that environment are important in cholera disease transmission. In addition, substantial levels of indirect vaccine protection, in addition to direct protection, could be attained if vaccine levels are high within an individual's social network and among neighbors sharing water resources.
